# West Nile virus methyltransferase domain interacts with protein kinase G

**DOI:** 10.1186/1743-422X-10-242

**Published:** 2013-07-22

**Authors:** Julie A Keating, Dipankar Bhattacharya, Pei-Yin Lim, Shaun Falk, Bernard Weisblum, Kristen A Bernard, Mayuri Sharma, Richard J Kuhn, Rob Striker

**Affiliations:** 1 Department of Medicine, W.S. Middleton Memorial Veteran’s Hospital, Madison, WI, USA; 2 Department of Medicine, University of Wisconsin-Madison, Madison, WI, USA; 3 Department of Pathobiological Sciences, School of Veterinary Medicine, University of Wisconsin-Madison, Madison, WI, USA; 4 Department of Biological Chemistry and Molecular Pharmacology, Harvard Medical School, Boston, MA, USA; 5 Department of Biological Sciences, Purdue University, West Lafayette, IN, USA

**Keywords:** Flavivirus, Phosphorylation, Interaction, Protein kinase G

## Abstract

**Background:**

The flaviviral nonstructural protein 5 (NS5) is a phosphoprotein, though the precise identities and roles of many specific phosphorylations remain unknown. Protein kinase G (PKG), a cGMP-dependent protein kinase, has previously been shown to phosphorylate dengue virus NS5.

**Methods:**

We used mass spectrometry to specifically identify NS5 phosphosites. Co-immunoprecipitation assays were used to study protein-protein interactions. Effects on viral replication were measured via replicon system and plaque assay titering.

**Results:**

We identified multiple sites in West Nile virus (WNV) NS5 that are phosphorylated during a WNV infection, and showed that the N-terminal methyltransferase domain of WNV NS5 can be specifically phosphorylated by PKG *in vitro*. Expressing PKG in cell culture led to an enhancement of WNV viral production. We hypothesized this effect on replication could be caused by factors beyond the specific phosphorylations of NS5. Here we show for the first time that PKG is also able to stably interact with a viral substrate, WNV NS5, in cell culture and *in vitro*. While the mosquito-borne WNV NS5 interacted with PKG, tick-borne Langat virus NS5 did not. The methyltransferase domain of NS5 is able to mediate the interaction between NS5 and PKG, and mutating positive residues in the αE region of the methyltransferase interrupts the interaction. These same mutations completely inhibited WNV replication.

**Conclusions:**

PKG is not required for WNV replication, but does make a stable interaction with NS5. While the consequence of the NS5:PKG interaction when it occurs is unclear, mutational data demonstrates that this interaction occurs in a region of NS5 that is otherwise necessary for replication. Overall, the results identify an interaction between virus and a cellular kinase and suggest a role for a host kinase in enhancing flaviviral replication.

## Background

The Flavivirus genus includes a number of medically-relevant arthropod-borne viruses, transmitted primarily by mosquito or tick. Mosquito-borne flaviviruses, such as yellow fever virus (YFV) and dengue viruses (DENV (1–4)), are transmitted primarily by *Aedes* mosquitoes [1,2], while West Nile virus (WNV) and Japanese encephalitis virus are transmitted primarily by *Culex* mosquitoes [3,4]. Tick-borne flaviviruses, including Langat virus (LGTV), constitute a distinct phylogenetic lineage and are poorly, if ever, transmitted by mosquitoes [5,6]. The *Flaviviridae* family, including the *Flavivirus* genus, consists of single-strand (+)-sense viruses. The genome is translated from a single open reading frame into a polyprotein, which is cleaved into separate structural and non-structural proteins. In flaviviruses, the non-structural protein NS5 is comprised of an N-terminal methyltransferase (MTase) domain [7,8] and a C-terminal RNA-dependent RNA polymerase (RdRp) domain [9]. The *Hepacivirus* and *Pestivirus* genera have NS5A and NS5B proteins; NS5B has RdRp activity [10].

A number of cellular kinases are able to phosphorylate viral proteins from a wide range of viruses, and the roles of kinases in viral lifecycles continue to be studied [11]. Serine/threonine phosphorylations of NS5 and NS5A are conserved throughout the *Flaviviridae* family [12] – phosphorylated NS5/NS5A is found in all three genera and in both mosquito- and tick-borne *Flaviviruses*[13,14]. The identity of many kinases able to phosphorylate flaviviral NS5, and the role of these kinases in viral infections, are unknown. We have previously demonstrated mammalian protein kinase G (PKG), a cGMP-dependent serine/threonine kinase, is able to phosphorylate dengue (DENV-2) NS5 at a site conserved among all mosquito-borne flaviviruses (Thr449, in the RdRp domain of NS5) [15]. Furthermore, DENV-2 NS5 is also phosphorylated by mosquito PKG, and alterations in PKG activity changes the flight behavior of *Aedes* and non-*Aedes* mosquitoes [16] raising the possibility that flaviviruses alter their own transmission from vectors.

In mammals, PKGI and PKGII are expressed from separate genes, and PKGI (referred to as PKG in this paper) is alternatively spliced at the extreme N-terminus into PKGIα and PKGIβ isoforms [17]. Both cGMP binding [18] and autophosphorylation [19] are involved in structural changes that induce PKG’s catalytic activity, increasing the enzyme’s ability to phosphorylate a substrate. Binding of cGMP to PKGI also induces a solvent-exposed enzymatic conformation that is hypothesized to allow for substrate binding to PKG [20]. Previous results showed that PKGIα, PKGIβ, and PKGII are all able to phosphorylate DENV-2 NS5 [15]. PKGIβ has previously been shown to increase replication of an HIV-1 LTR-reporter [21], but a specific role for PKG in viral lifecycles has not been demonstrated.

In this paper, we have expanded our study of PKG’s role in flaviviral infections from *Aedes*-borne DENV to *Culex*-borne WNV. We identified multiple phosphorylation sites in WNV NS5 and report that a truncated WNV NS5 (MTase only), without the previously identified NS5 phosphorylation site in DENV, is also a substrate for phosphorylation specifically by PKG. The Ser38 site in the MTase domain is phosphorylated during a WNV infection. Additionally, we showed that WNV NS5 is able to form a stable interaction with human PKGIβ. Tick-borne LGTV NS5 did not interact with PKG, suggesting further substrate-specificities of PKG for mosquito- versus tick-borne flaviviral NS5. The MTase domain alone of WNV NS5 interacted with PKG, and mutating specific residues involved in the interaction rendered the WNV replicon non-functional. This is the first demonstration that the αE helix of NS5 is critical for WNV replication and that PKG binding occurs at this critical region, even though PKG itself is likely not required for viral replication.

## Results

### WNV NS5 is phosphorylated in infected HEK293T cells

**Figure 1:**
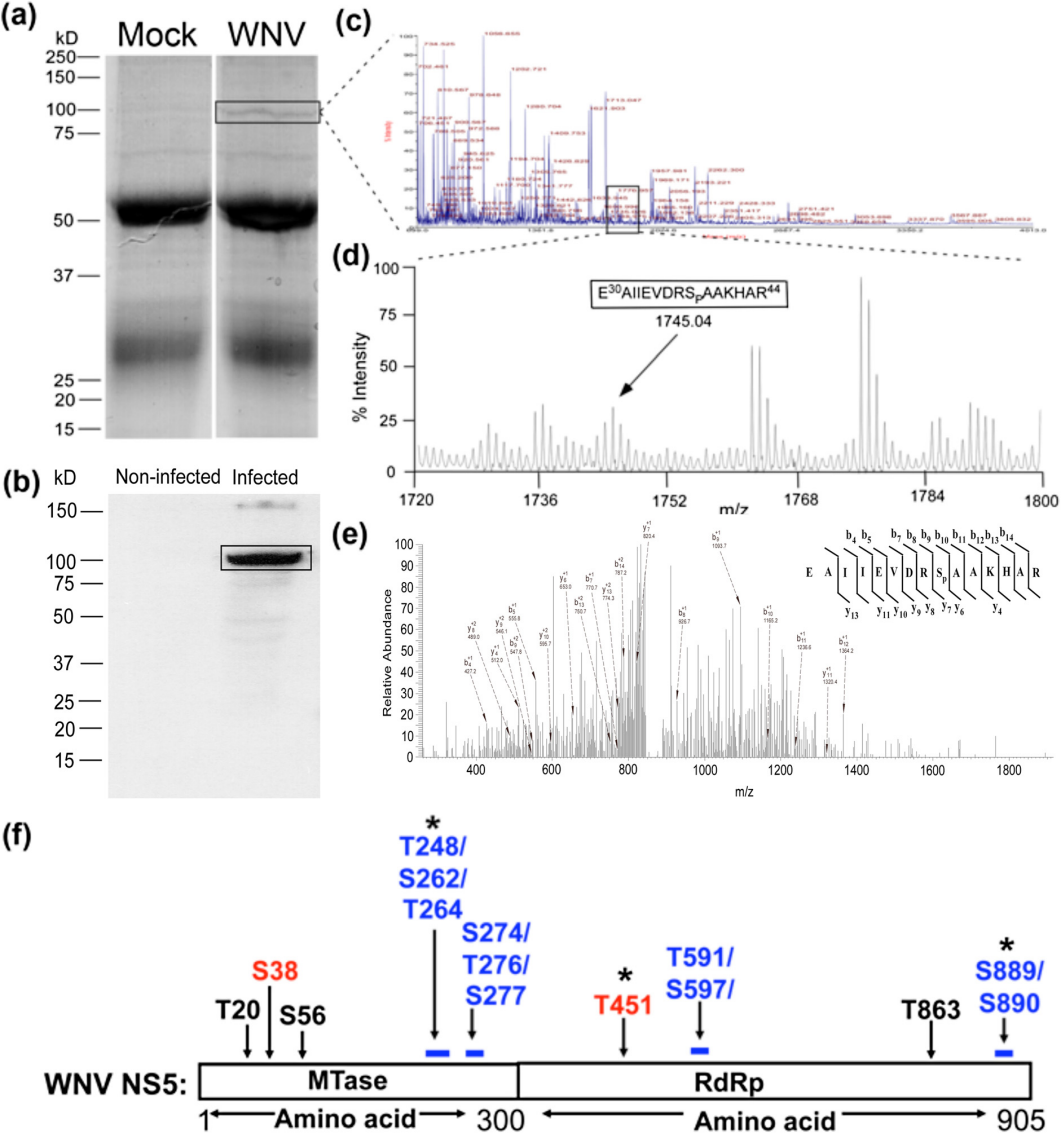
**Multiple sites in NS5 are phosphorylated during a WNV infection.** HEK293T cells were infected with buffer (Mock) or WNV (MOI = 10), harvested at 24 hpi, and subjected to immunoprecipitation with α-WNV MTase antibody. **(a)** The elution was subjected to SDS-PAGE analysis. The 100 kD band corresponding to WNV NS5 in infected cells was excised for mass spectrometric analysis. **(b)** Western blot using α-WNV MTase antibody after immunoprecipitation identified the 100 kD band isolated from the infected cells as WNV NS5. **(c)** Spectrum of full scan from the MALDI-TOF of the 100 kD band identified the protein sample as WNV NS5. **(d)** Enlarged spectrum showing the peaks boxed in C. Arrow points to a singly charged monoisotopic peak of a single phosphorylated peptide corresponding to aa 30–44 with a mass of 1745.04 Da, which includes 79.9 Da corresponding to one phosphorylation. **(e)** Liquid chromatography tandem mass spectrometry (LC-MS/MS)Data of the phosphorylated peptide Glu30-Arg44 showed a phosphorylation on Ser38. The peptide was purified using a C-18 high performance liquid chromatography (HPLC) column before fragmentation. The mass was calculated for post-translational modifications with an addition of 79.9 for each phosphate. Major fragment ions are labeled with their corresponding b (C-terminal) and y (N-terminal) ions and their charge state. **(f)** The schematic shows the individual serine or threonine residues present in the WNV NS5 phosphopeptides identified by MALDI-TOF mass spectrometry. Ser38 and Thr451 (in red) were phosphorylated by PKG *in vitro*. Residues shown in black were present in phosphopeptides with a single serine or threonine. Phosphopeptides with one phosphosite, but more than one serine or threonine residue that may be phosphorylated, are shown in blue. Further analysis is necessary to determine exactly which serine or threonine in these phosphopeptides is phosphorylated. Asterisks indicate the presence of Tyr residues in the phosphopeptide.

To identify specific sites that are phosphorylated in WNV NS5 during an infection, HEK293T cells were infected with WNV at a multiplicity of infection (MOI) of 10, and cell lysates were harvested at 24 hours post-infection (hpi). WNV NS5 was immunoprecipitated (IP) with a polyclonal antibody to WNV MTase, and the eluted proteins were subjected to SDS-PAGE analysis (Figure 1(a)). A Western blot with polyclonal antibody against WNV MTase confirmed the presence of NS5 in WNV-infected, but not in mock-infected, HEK293T cell lysate (Figure 1(b)). The ~100 kD band corresponding to full-length NS5 (amino acids (aa) 1–905) was excised from the gel (Figure 1(a)) and treated with trypsin. One half of the peptide sample was treated with phosphatase to remove phosphates and was used as a reference for mass comparisons with the untreated sample (data not shown). Both the phosphatase-treated and untreated samples were subjected to MALDI-TOF analysis (Figure 1(c) and (d)). A phosphopeptide with a mass of 1745.04 Da, corresponding to aa Glu30-Arg44, was identified pi (Figure 1(d)). The same peptide was also detected in the phosphatase-treated sample with a mass of 1667.03Da, which corresponds to the loss of one phosphate (data not shown). Tandem mass spectrometry of the untreated sample also confirmed the phosphorylation of the Glu30-Arg44 peptide, and specifically, the phosphorylation of Ser38, in NS5 MTase during a WNV infection (Figure 1(e)). A phosphoacceptor at Ser38 is conserved among many, though not all, mosquito-borne flaviviral NS5 proteins (data not shown). In addition to Ser38, eight other putative phosphopeptides were found in WNV NS5 (including a peptide containing Thr451, the site conserved with Thr449 of DENV NS5) during a WNV infection (Figure 1(f)).

### The WNV MTase domain is a major substrate for PKG phosphorylation

**Figure 2:**
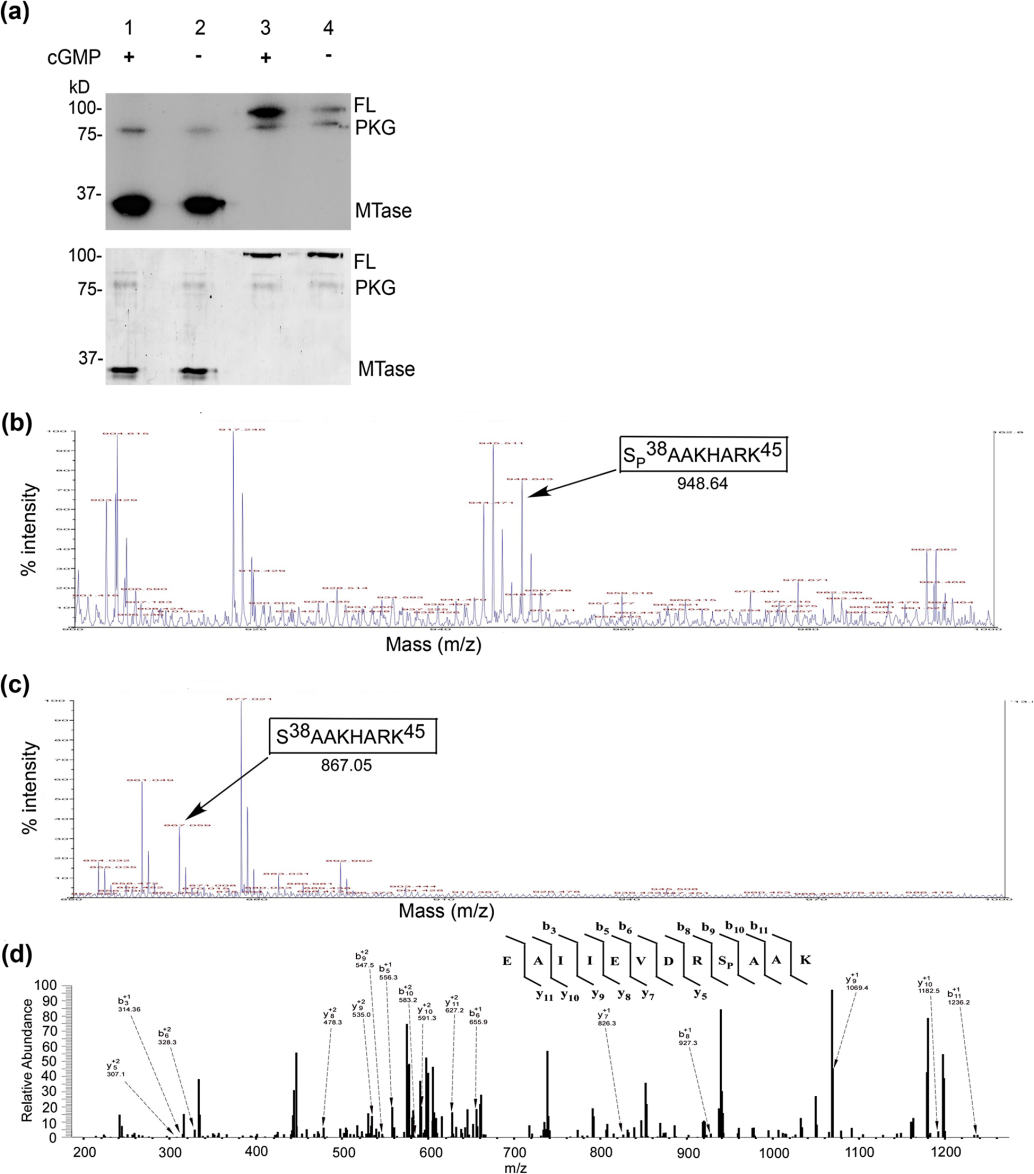
**WNV MTase domain is phosphorylated at Ser38 by PKG*in vitro*. (a)** WNV MTase and full-length (FL) NS5 were bacterially expressed and purified. MTase (Lane 1 and 2) and full-length NS5 (Lane 3 and 4) were used as substrates in an *in vitro* kinase assay with PKGIα enzyme and γ-^32^P ATP. The upper panel is an autoradiograpHshowing relative levels of ^32^P incorporation, indicative of protein phosphorylation. The lower panel is the same SDS-PAGE gel, Coomassie-stained to demonstrate equal protein loading. **(b)** Bacterially expressed and purified WNV MTase was incubated with bovine lung PKGIα, cGMP, and non-radiolabelled ATP. The sample was separated on 10% SDS-PAGE, and the 33kD band corresponding to the MTase was excised for mass spectrometric analysis. The sample was subjected to an in-gel tryptic digest, and half of the sample was treated with 20 units of phosphatase. The MALDI-TOF spectrum of the sample that was not treated with phosphatase is shown, with the monoisotopic peak of the singly phosphorylated peptide corresponding to aa Ser38-Lys45 with a mass of 948.64 Da indicated. **(c)** MALDI-TOF spectrum of the sample after treatment with phosphatase. The peak corresponding to the Ser38-Lys45 peptide is shown with an approximately 80 Da decrease in mass, as compared to **(b)**. **(d)** LC-MS/MS ionizations of the phosphorylated peptide corresponding to aa Glu30-Lys41 showing the specific phosphorylation of the Ser38 residue. The peptide was further purified through a C-18 HPLC column before fragmentation. The mass was calculated for post-translational modifications with an addition of 79.9 for one phosphate in the peptide sequences. Major fragment ions are labeled with their corresponding b (C-terminal) and y (N-terminal) ions and their charge state.

HEK293T cells have endogenous PKG activity (data not shown), so we examined whether PKG could be responsible for the phosphorylations identified in WNV-infected HEK293T cells. To determine if WNV NS5 and/or the WNV MTase domain alone (aa 1–300) were substrates for PKG phosphorylation, WNV NS5 full-length and MTase proteins were bacterially expressed and purified. The proteins were used as substrates in an *in vitro* kinase assay with PKGIα kinase as described above. An autoradiograpH (Figure 2(a), upper panel) shows that while full-length NS5 is phosphorylated by PKG (lanes 3 and 4), the MTase domain alone (lanes 1 and 2) was also phosphorylated in the presence of PKG. The WNV NS5 Thr451 site, conserved with the DENV Thr449 PKG phosphoacceptor, is present outside of the MTase domain. There was greater phosphorylation of both MTase and full-length NS5 substrates and greater autophosphorylation of PKG in the presence of cGMP, supporting that NS5 is a typical PKG substrate with phosphorylation enhanced in the presence of cGMP. A similar *in vitro* experiment also showed PKG phosphorylation of DENV MTase, and there was an approximately three-fold increase in PKG phosphorylation signal for DENV-2 MTase and WNV MTase as compared to LGTV MTase (data not shown). These results suggest that MTase phosphorylation by PKG is, again, more specific to mosquito- than tick-borne flaviviruses.

To identify specific residue(s) phosphorylated by PKG, an *in vitro* kinase reaction using WNV MTase domain and PKGIα was performed. The reaction was subjected to SDS-PAGE analysis, and the gel band corresponding to WNV MTase (33 kD) was excised and in-gel trypsin-digested. Half of the sample was treated with phosphatase. Both the phosphorylated and dephosphorylated samples were analyzed with MALDI-TOF mass spectrometry. Results from MALDI-TOF analysis identified a specific peptide corresponding to aa Ser38-Lys45 in the MTase domain (Figure 2(b)), which lost ~80 Da in mass (equivalent to a phosphate group) after phosphatase treatment compared to the untreated peptide (Figure 2(c)). These results support our hypothesis that the greater mass of the Ser38-Lys45 peptide in the non-phosphatase-treated sample was due to the presence of a phosphate group on Ser38. Subsequent MS/MS analyses also confirmed that the Ser38 residue of WNV MTase was phosphorylated specifically by PKG *in vitro* (Figure 2(d)). Subsequent mutational analysis of the 38 residue suggested that WNV was still able to replicate even when it was unphosphorylatable at Ser38 in WNV replication (data not shown), thus we shifted our focus to a more overall role for PKG in WNV infections.

### WNV viral production is enhanced in BHK-21 cells expressing PKG

**Figure 3:**
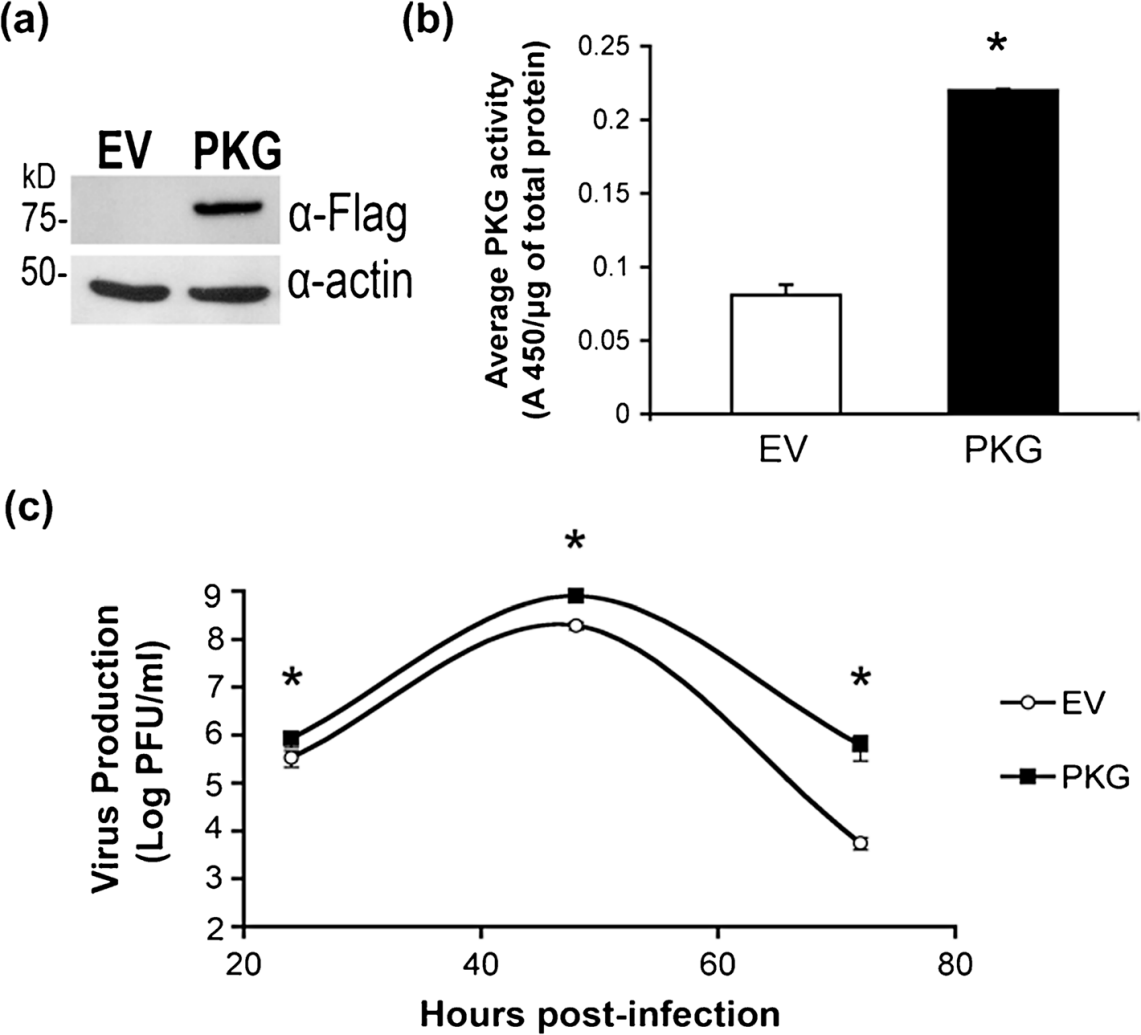
**PKG expression is associated with an enhancement in viral production. (a)** Expression of PKG in stably transfected BHK-21 cells containing the empty vector (BHK + EV, labeled as EV) or vector expressing PKG (BHK + PKG, labeled as PKG). Cell lysates were subjected to Western blot analysis using α-Flag and α-actin. Western blots show the expression of PKG in BHK + PKG, but not BHK + EV, stable cell lines. **(b)** The relative level of PKG activity in each BHK stable cell line. PKG activity was assayed in each stably transfected cell lysate by measuring the phosphorylation of a canonical PKG peptide substrate. **(c)** Effect of PKG on WNV production. The stably transfected BHK cell lines were infected with WNV. Viral production in PFU/ml was measured in quadruplicate by plaque-forming assay at various time-points pi. The figure is a representative experiment of one of three independent experiments. Significant *P*-values between BHK + EV and BHK + PKG are indicated as follows: *, *P*< 0.05.

It is well-known that WNV replicates in BHK-21 cells, which we confirmed to have undetectable PKG activity [22]. While we found that Ser38 is phosphorylated during an infection and by PKG *in vitro*, PKG itself may not be required for viral replication. To determine the overall effect of PKG on WNV replication, a stable BHK-21 cell line expressing PKGIβ-Flag (BHK + PKG) and a BHK-21 stable cell line containing the empty vector (BHK + EV) were generated. We confirmed the expression of PKG in BHK + PKG cells, but not in BHK + EV cells (Figure 3(a)). A PKG activity assay showed that BHK + PKG cells had significantly higher PKG activity than BHK + EV cells (Figure 3(b)), indicating that BHK + PKG cells express catalytically active PKG. We infected both BHK stable cell lines with WNV to determine if PKG expression enhances WNV production during an infection. Both cell lines were infected with WNV (MOI = 0.5), and virus production at 24, 48, and 72 hpi was measured by plaque assay. WNV production was enhanced two- to ten-fold in the BHK + PKG cell line compared to BHK + EV cells (*P* = 0.04 at 24 hpi, *P* = 0.03 at 48 hpi, and *P* = 0.03 at 72 hpi) (Figure 3(c)). Similar experiments with a DENV infection found that DENV production was also five- to ten-fold greater in BHK + PKG cells as compared to BHK + EV cells (Additional file 1: Table S1), suggesting a conserved effect of PKG on mosquito-borne flaviviruses.

### WNV NS5, but not LGTV NS5, interacts with mammalian PKGIβ

**Figure 4:**
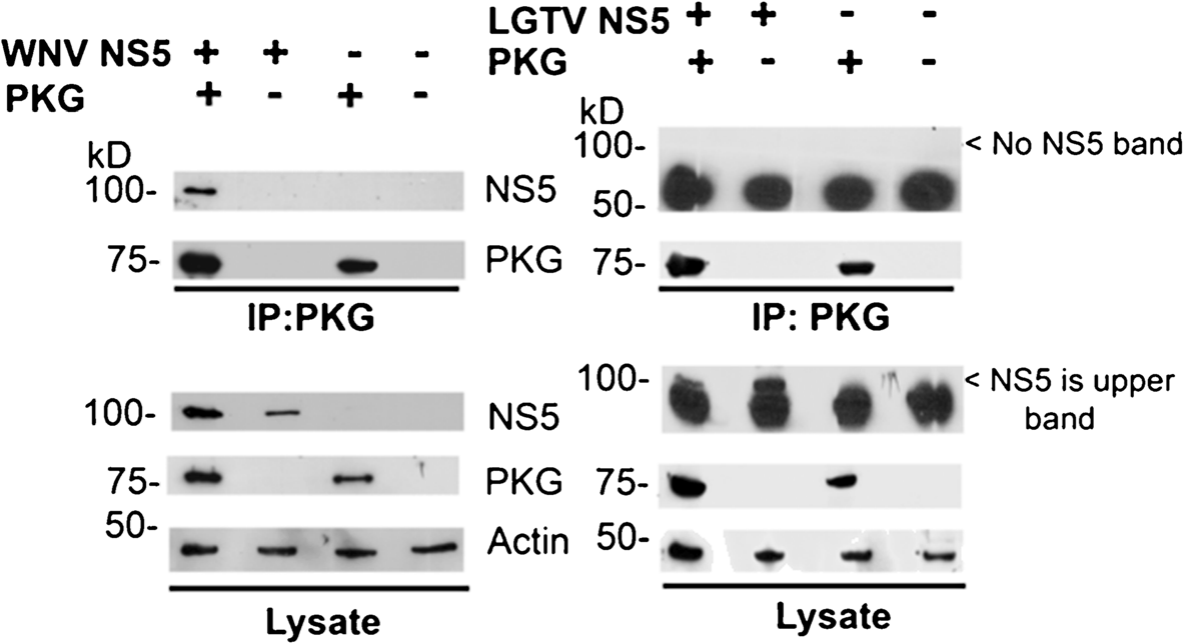
**WNV NS5, but not tick-borne LGTV NS5, interacts with PKGIβ.** HEK293T cells were transfected with DNA plasmids encoding WNV NS5 or LGTV NS5 and PKGIβ-Flag. Total protein from the lysate was subjected to immunoprecipitation using α-Flag. Total cell lysates or immunoprecipitated proteins were subjected to Western blot analysis using α-WNV MTase (for the detection of WNV NS5 (left panel)) and α-HA (for the detection of LGTV NS5 (right panel)), α-Flag (for the detection of PKG), and α-actin to show equal protein loading for the lysate.

How PKG is enhancing WNV replication is unknown. We hypothesized that WNV NS5 and PKG could interact, providing a potential cause for enhancement. HEK293T cells were co-transfected with plasmids expressing WNV NS5 and human PKGIβ-Flag, and cells were lysed at 48 hours post-transfection. We immunoprecipitated PKG using monoclonal antibodies against Flag and performed Western blot analysis to test for the presence of WNV NS5. NS5 co-immunoprecipitated with PKG in the co-transfected cells, but not in empty vector or mock-transfected cells (Figure 4, left panel). NS5 was not found in any control co-immunoprecipitations. When the co-immunoprecipitation experiment was performed with LGTV NS5 and PKG, LGTV NS5 did not co-immunoprecipitate with PKG (Figure 4, right panel).

### The WNV MTase domain can interact with PKG

**Figure 5:**
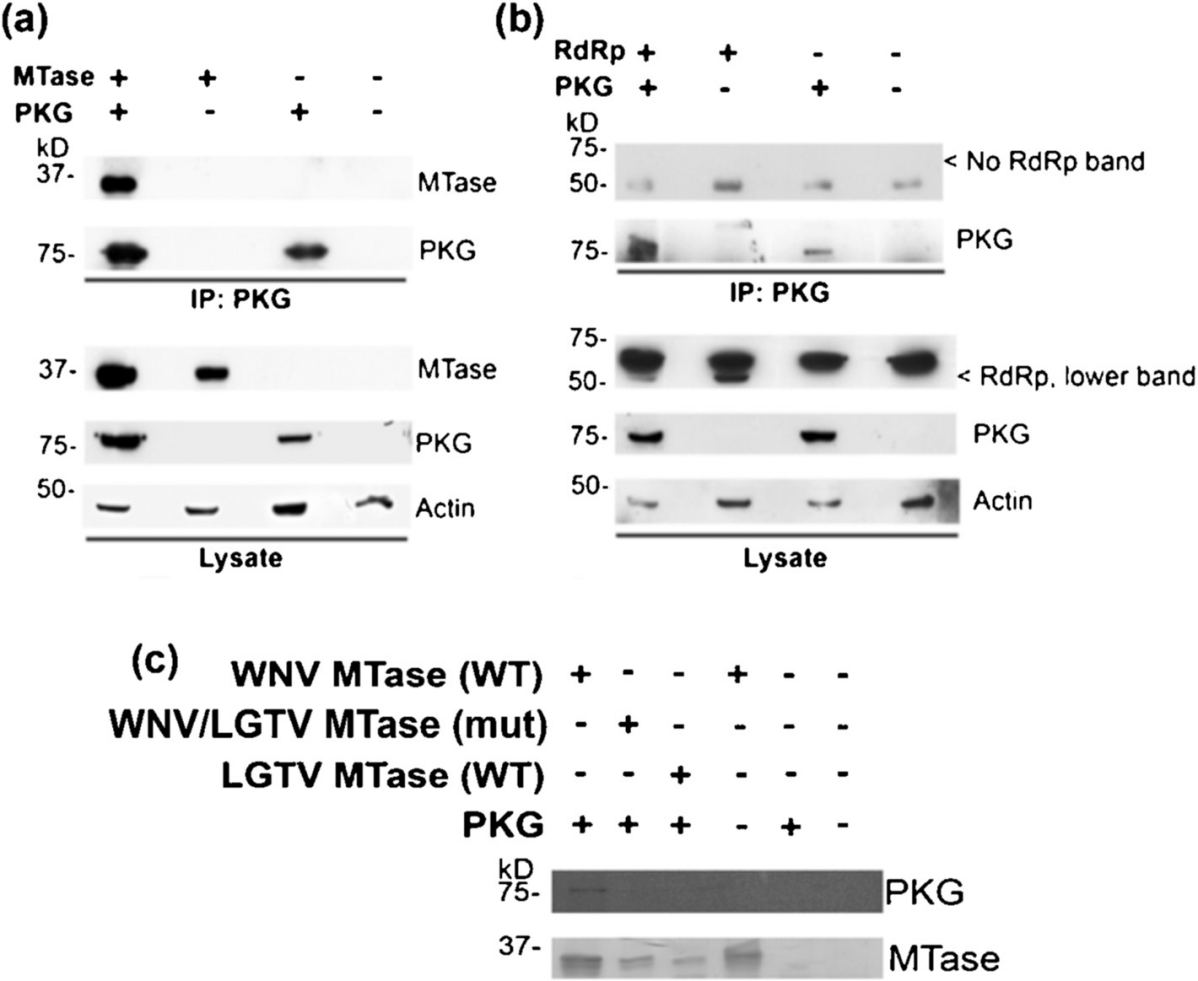
**The MTase domain interacts with PKGIβ through the αΕ helix.** HEK293T cells were transfected with plasmid encoding wild-type PKGIβ-Flag and **(a)** WNV MTase or **(b)** WNV RdRp. Cell lysates were subjected to immunoprecipitation using α-Flag. The cell lysates and immunoprecipitated proteins were subjected to Western blot analysis using α-actin, α-WNV MTase, α-Flag (PKG), or α-HA (RdRp). **(c)** WNV MTase (wild-type (WT) or chimeric mutant with LGTV sequence at αΕ (Mut)) and LGTV MTase (WT) were purified onto Ni-NTA beads and incubated with ^35^S-labeled human PKGIβ-Flag in an *in vitro* binding assay. Beads were washed, and eluted proteins were separated by SDS-PAGE. An autoradiograpH(upper panel) shows PKG that precipitated with MTase. The lower panel is the Coomassie-stained SDS-PAGE gel.

The MTase domain alone (in WNV, aa 1–300) is a substrate for PKG phosphorylation (Figure 2). To determine if the MTase domain alone is sufficient to interact with mammalian PKG, cells were co-transfected with plasmids expressing WNV NS5 MTase and human PKGIβ-Flag. The co-IP experiment was performed as described above. The WNV MTase domain (Figure 5(a)), but not the RdRp domain alone (aa 301–906, Figure 5(b)), co-immunoprecipitated with PKG in the co-transfected cells. These results suggest that the interaction site for PKG is present within the MTase domain of WNV NS5.

Upon finding that the MTase domain alone was able to interact with PKG, we began to search for the specific region(s) within the MTase domain that were involved in the interaction. A primary region of interest was the αE helix in the MTase (aa M187-G202). Mutagenesis of the αE helical region in the DENV MTase previously suggested that this helix interacts with DENV NS3 protein [23]. The region was fairly well-conserved among mosquito-borne flaviviruses, but differed at a few distinct residues from the complementary sequence in tick-borne LGTV NS5 (Table 1). To determine if this region as a whole was involved in WNV MTase’s interaction with PKG, the WNV sequence for aa187-202 was replaced with the LGTV sequence at the same region within the MTase. Because LGTV NS5 did not interact with PKG, we hypothesized that substituting the LGTV αE region into the WNV MTase would interrupt the interaction between WNV MTase and PKG. In fact, co-immunoprecipitation assays showed that this chimeric mutant WNV MTase with LGTV αE sequence did not interact with PKG; unfortunately, mutant MTase expressed poorly compared to wild-type (WT) in these assays (data not shown). We utilized an *in vitro* binding assay, which confirmed that while WT WNV MTase interacted with PKG, the WNV/LGTV MTase chimeric mutant did not interact with PKG (Figure 5(c)). These *in vitro* results also confirm that LGTV MTase does not interact with PKG.

**Table 1:** αE sequence in flaviviruses

**Virus (GenBank accession number)**	**αE sequence (aa187-202)**
WNV (AAV68177)	MPKVIEKMELLQRRYG
WNV αE	MPKAIEAMELLQAAYG
DENV-1 (ACW82891)	MPSVVETLEQMQRKHG
DENV-2 (AAA73185)	MPSVIEKMEALQRKYG
DENV-3 (AAS49480)	MPTVIEHLERLQRKYG
DENV-4 (AEW51803)	MPTVIEELEKLQRKHG
YFV (AAC35901)	MPDVLEKLELLQRRFG
LGTV (AAF75260)	RPEVIEALHRFQLQWG

### Residues within the αE helix region of WNV MTase are involved in MTase’s interaction with PKG

**Figure 6:**
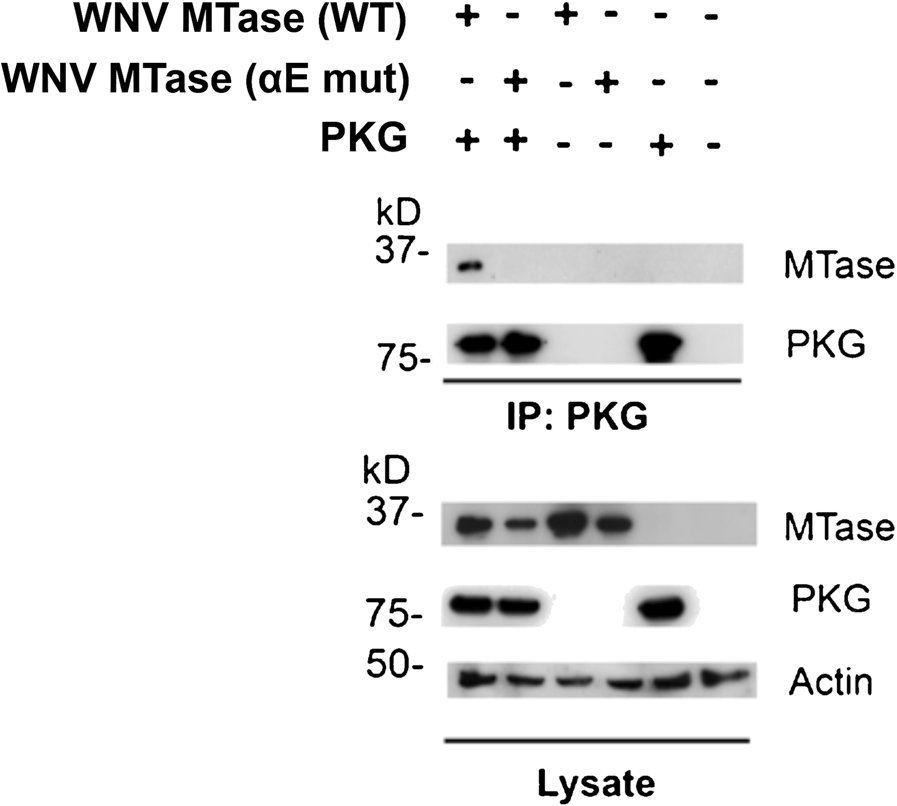
**Mutating positive residues in the MTase αE helix prevents WNV MTase’s interaction with PKG.** In the WNV MTase, Lys189, Lys193, Arg199, and Arg200 (Table 1) were mutated to Ala to create WNV MTase (mut). HEK293T cells were transfected with plasmids encoding WNV MTase (WT) or WNV MTase (mut) and PKGIβ-Flag or were mock transfected. PKGIβ was immunoprecipitated using α-Flag, and the presence of WNV MTase in co-immunoprecipitation and lysate samples was detected by Western blot with α-WNV MTase. PKG and actin were detected by Western blotting with α-Flag and α-actin, respectively.

To further identify specific residues within the αE helix responsible for the interaction, we mutated four amino acids that had charge changes between WNV and LGTV sequences. In the WNV MTase (mut) construct, Lys189, Lys193, Arg199, and Arg200 were all mutated to Ala (indicated in red in Table 1). Again, this mutant WNV MTase, unlike WT, did not interact with PKG in a co-immunoprecipitation assay (Figure 6). Thus, these four residues within the αE helix appear to be involved in WNV MTase’s interaction with PKG.

### αE mutations inhibit WNV replication

**Figure 7:**
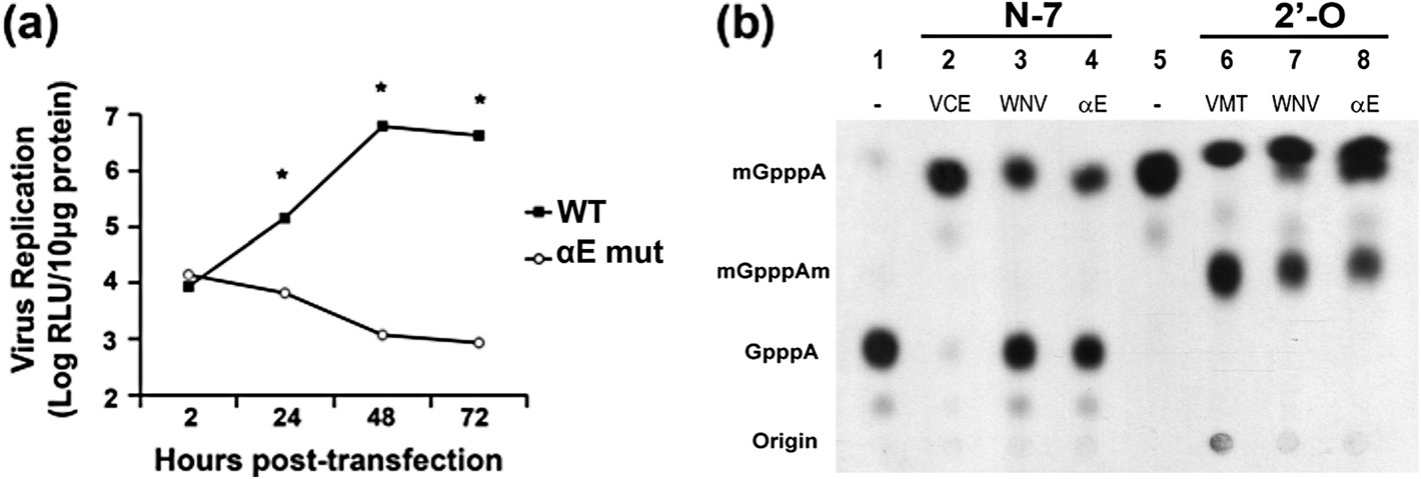
**αE mutations completely inhibit WNV replication. (a)** HEK293T cells were electroporated with wild-type (WT) or αE mutant (αE Mut) WNV RLuc2A replicon RNA. Cells were lysed at indicated time points post-transfection, and the total luciferase activity in 10 μg total protein from cell lysate was measured in relative light units (RLUs) by luciferase assay. Each sample was read in triplicate, and figure is representative of two experimental replications. Statistical significance is represented as follows: *, *P*< 0.0005. **(b)** Capped WNV RNA corresponding to the first 190 nucleotides of the WNV genome was used to examine both N-7 (lanes 2–4) and 2′-O (lanes 6–8) methylation. For N-7 methylation, capped WNV RNA was incubated in the presence of SAM with either mock (1), Vaccinia virus capping enzyme (VCE) (2), wild-type WNV methyltransferase (3), or WNV αE mutant methyltransferase (4). For 2′-O methylation, m7GpppA-WNV RNA was incubated in the presence of SAM with mock (5), Vaccinia 2′-O methyltransferase (VMT) (6), wild-type WNV methyltransferase (7) or WNV αE mutant methyltransferase (8). The reactions were run out by TLC on PEI cellulose and ability of αE mutant WNV methyltransferase to generate both m7GpppA and m7GpppAm was compared to wild-type WNV.

To determine whether interrupting the interaction between WNV NS5 and PKG would have an effect on WNV replication, the same four αE mutations (indicated in red in Table 1) were introduced into the WNV replicon. WT and αE mutant replicon RNA was electroporated into HEK293T cells, and cells were lysed at various time point post-transfection. The relative light units (RLU) in 10μg total protein from cell lysate were measured by luciferase assay. While both WT and αE mutant (αE Mut) samples had comparable input luciferase readings (2 hours post-transfection), the mutant replicon failed to replicate (Figure 7(a)).

Residues within the αΕ region have not been identified as involved in MTase’s N-7 or 2’-O methylation reactions. However, we wanted to ensure that our introduction of mutations was not inhibiting MTase activity to induce the loss of replication. Using a 190 base pair capped RNA as a substrate we compared the N7 and 2’0 MTase activity of recombinant WNV MTase with that of the four αΕ mutant WNV MTase. No clear defect in MTase activity was seen (Figure 7(b)).

## Discussion

While WNV NS5 has been previously shown to be phosphorylated [24], the specific sites have not been previously mapped. The MTase domain is an effective substrate for PKG phosphorylation, and is phosphorylated specifically at the Ser38 site during a WNV infection and by PKG *in vitro*. Additionally, WNV NS5 forms a stable interaction with mammalian PKG. Specific charged residues within the αE helix of the MTase domain are able to mediate this interaction, and mutating these same residues prevents WNV replication.

When PKG is present in cells, there is a stable interaction between WNV NS5 and PKG. Stable interactions between a cellular kinase and viral substrate are not unprecedented [25], and in fact the hepatitis C virus protein NS5A, related to WNV NS5, is able to co-immunoprecipitate with cellular kinase phosphatidylinositol 4-kinase type IIIα from cell culture [26]. It is unknown why this, or any, kinase might have a more stable interaction - it is possible that the kinase’s association with the viral protein has an effect beyond the substrate phosphorylation itself. Additional insight into the role of PKG in flaviviruses may require the development of an NS5 that is both replication competent and unable to interact with PKG.

We have shown that mutating the positive residues in the αE region that are involved in the NS5-PKG interaction prevents replication of the WNV replicon. As multiple mutations were added to the WNV replicon, it is unclear if factors other than WNV NS5-PKG interaction have been disrupted. For example, the literature suggests that several charged residues (mostly Glu) in the αE region are involved in the interaction between DENV-2 NS5 and NS3 [23]. We changed four basic residues in the same region disrupting the MTase-PKG interaction, but possibly also disrupting the NS5-NS3 interaction or other functions. These mutations did not dramatically alter the MTase function of NS5. While we do not yet have a specific mechanism for the role of the NS5-PKG interaction, ourData do show that the αE region is important for WNV replication and that PKG utilizes this important region for its interaction.

Flaviviruses replicate well in BHK-21 cells, which are naturally PKG-deficient [22], thus it seems that PKG is not necessary for propagation of flaviviruses in cell culture. When phosphorylation of flaviviral NS5 (DENV-2) was first detected by relatively unsensitive techniques, it was particularly prominent only under low serum conditions [13]. However, the NS5 protein from multiple mosquito-borne flaviruses (WNV (above), DENV-2 [15], and YFV [27]) have now been shown to be a substrate for PKG, and we show here that PKG’s expression in BHK-21 cells is associated with an enhancement in WNV replication. As flaviviruses have limited coding ability and high replication rates, the conservation suggests an evolutionary advantage for the preservation of PKG substrate sites. Furthermore, activation of PKG in mosquitos leads to increased wing activity [16], similar to the increase in foraging noted in insects that are not viral disease vectors [28,29]. This could be the mechanistic basis for the recently reported increase in mosquito flight caused by DENV [30] and could facilitate viral transmis-sion and account for the conservation of PKG phosphorylatable sites in mosquito-transmitted flaviviruses.

## Conclusions

We show that mosquito-borne, but not tick-borne, flaviviral NS5 are substrates for phosphorylation by and interact with mammalian PKG. The data here suggest further substrate specificities for mammalian PKG between mosquito- and tick-borne flaviviral NS5, as LGTV NS5 does not interact with PKG as WNV NS5 does. The WNV MTase domain alone is able to interact with PKG, so this domain is a significant substrate for both PKG phosphorylation and interaction. The αE helical region of the MTase domain is involved in the interaction between WNV NS5 and PKG. Specific mutations within this region that interrupt the WNV MTase-PKG interaction result in non-functional WNV replication. While a specific mechanism for PKG’s phosphorylation of and interaction with NS5 affects flaviviral replication remains unknown, the results provide more information about an overall role for PKG in both *Aedes-* and *Culex*-borne flaviviral infections.

## Materials and methods

All research was approved by the relevant committees at UW Madison.

### Cell culture and viral infections

Human embryonic kidney (HEK293T, ATCC#CRL-11268) and baby hamster kidney cells (BHK-21; ATCC #CCL-10) cells were grown in Dulbecco’s Modified Eagle Medium (DMEM, Sigma, St Louis, MO) supplemented with 10% heat-inactivated fetal bovine serum (HI-FBS), 100 U/mL penicillin and 0.1 mg/mL streptomycin (Sigma). African green monkey kidney cells (Vero; ATCC #CCL-81) were maintained in minimal essential medium (MEM, Invitrogen, Carlsbad, CA) supplemented with 10% HI-FBS, 2 mML-glutamine and 1.5 g/L sodium bicarbonate, 100 U/mL penicillin, and 100 μg/mL streptomycin. Cells were incubated in 5% CO_2_ at 37°C.

WNV was produced from an infectious cDNA clone of an isolate from New York in 2000 [31]. RNA was *in vitro* transcribed from the cDNA clone and then electroporated into BHK-21 cells as previously described [32]. Viral titers were determined by plaque assays on Vero cell monolayers. For WNV infections, HEK293T were seeded and incubated at 37°C overnight. Medium was removed, and the cells were inoculated with WNV in DMEM medium supplemented with 1% HI-FBS at 37°C. At 1hour post-inoculation, the medium was replaced with DMEM supplemented with 10% HI-FBS, 50 U/mL penicillin, and 0.05mg/mL streptomycin, and the cells were incubated at 37°C. Supernatants were harvested at the indicated time points post-infection and stored in aliquots for titering by plaque assay on Vero cells. For mass spectrometry analysis, infected HEK293T cells were lysed by homogenization in Tris-Cl buffer containing 1% NP-40, 1X Halt Inhibitor Protease Cocktail (Pierce, Rockford, IL) and 1X Halt Phosphatase Inhibitor Cocktail (Pierce). WNV NS5 was purified from the cell lysate by immunoprecipitation overnight with a custom-made rabbit polyclonal antibody against WNV NS5 (Covance, Princeton, NJ).

### Mass spectrometry sample preparation, loading, and analysis

WNV NS5 (full length or MTase domain only) protein samples were prepared for mass spectrometry andData analysis as described previously [15]. In short, WNV NS5 immunoprecipitated protein from HEK293T cells (in case of viral infection) or *E. coli*-expressed Ni-NTA affinity purified WNV MTase domain was subjected to run in 10% SDS-PAGE gel. After the targeted band was excised from gel (identified with GelCode Blue stain (ThermoFisher Scientific, MA)), in-gel digestion with Trypsin Gold (Promega, WI) in combination with Proase MAX Surfactant (Promega, WI) were performed at 50°C for 90minutes. After peptide recovery from gel pieces one half of the sample was treated with 20 units of calf intestinal alkaline phosphatase (CIAP) for dephosphorylation at 37°C for two hours. Both phosphorylated and dephosphophorylated peptides were then desalted with C18 Zip-Tip column (Millipore, MA) and run in both MALDI-TOF/TOF (Applied Biosystem, CA) and Finnigan LTQ Linear Ion Trap mass spectrophotometer (Thermo-Fisher Scientific, MA) for LC-MS/MS at University of Wisconsin-Madison Mass Spec Facility Center as described previously [27]. MALDI-TOFDatawere collected from the range of m/z 600 and 4000 and the spectra analysis was performed in GPS Explorer software (Applied Biosystem, CA). MascotDatabase (Matrix Science, UK) search andData comparison before and after CIAP treatment, detailed matching of mass with peptide identification was performed in a web-based software in Protein Prospector (v 4.27.1) called MS-Digest tools. ESI precursor ion scan mass spectra were obtained using Finnigan LTQ Linear Ion Trap mass spectrometer equipped with a nanoelectrospray ion source. Zorbax 300SB-C18 nanoflow HPLC column (150 mm × 75 μm, Agilent Technologies, Santa Clara, CA) was eluted with a linear gradient of water-acetonitrile in the presence of 0.1% formic acid at a flow rate of 0.2 μl/min. The remainder of the protocol was followed as described previously [33] and full scan spectra were recorded from m/z 200–2000 followed by MS/MS spectra in Xcalibur software (ThermoFisher Scientific, MA). MS/MS spectra of detected phosphopeptides were searched against the FASTADatabase using the Sequest search program within BioWorks Rev3.3 software (ThermoFisher Scientific, MA).

### DNA constructs and cloning

The full-length WNV NS5 was amplified from a 6XHis-tagged WNV NS5 in pET-LIC template by PCR (forward primer 5′-GCTAGCATGGCCGGTGGGGCAAAAGGACGC-3′ and reverse primer 5′-TTAATTAACAGTACTGTGTCCTCAACCAAAG-3′) and cloned into a 6XHis IRES GFP vector [15] using NheI and PacI. The WNV MTase domain (aa 1–300) was amplified from the full-length clone using the forward primer 5′-GCTAGCATGGCCGGTGGGGCAAAAGGACGC-3′ and reverse primer 5′-TTAATTAAGTTCTCATCGTGGTGCCACGTCG-3′ and cloned into the IRES GFP vector using NheI and PacI. The RdRp domain (aa 301–906) was amplified to include a 5′ HA tag with the forward primer 5′-ATGTACCCATACGATGTTCCAGATTACGCTCACCCATATAGAACC-3′ and reverse primer 5′-TTAATTAACAGTACTGTGTCCTCAACCAAAG-3′ and cloned into the IRES GFP vector using NheI and PacI. WNV MTase was cloned into the pQE-30 bacterial expression system (Qiagen, Germantown, MD) with BamHI and HindIII for protein expression and purification. Full-length LGTV NS5 (aa 1–904) was cloned into the IRES GFP vector with an HA tag, using NheI and PacI.

C-terminally Flag-tagged human PKGIβ was obtained from Dr. Kuan-TehJeang [21], and the full-length tagged gene was cloned in the pCI-Neo vector using NotI and XhoI. The resulting PKGIβ-Flag construct was used in DNA transfections.

The pACYC WNV RLuc2A Replicon has been previously described [34]. The αE region was mutated by PCR amplifying the SpeI-XbaI fragment (nucleotide 6771–9975), cloning into the TOPO vector (Invitrogen, Carlsbad, CA), and performing site-directed mutagenesis using QuikChange Site-Directed Mutagenesis Kit (Stratagene, La Jolla, CA). The mutated fragment was then cloned back into the replicon vector.

### Expression and purification of proteins

His-tagged WNV MTase was expressed in M15/pREP4 *E. coli* (Qiagen) using the pQE-30 bacterial expression system. Protein expression was induced by incubating *E. coli* with 1 mm isopropyl-β-D-thiogalactopyranoside (Invitrogen) for 7 hours. Bacterial cells were collected by centrifugation and lysed by sonication in buffer containing 50 mm Tris (pH8.0), 300 mm NaCl, and 10 mm imidazole. Cleared lysate was incubated with Ni-NTA beads (Qiagen) for His-tag binding, and bound protein was eluted in buffer containing 50 mm Tris (pH8.0), 300 mm NaCl, and 250 mm imidazole.

### *In vitro* kinase assay

Each *in vitro* kinase reaction was performed by incubating 1 μg of purified WNV NS5 (full-length or MTase domain only) substrate with 100 ng bovine lung PKGIα (Calbiochem, Gibbstown, NJ), 5 μCi of γ-P^32^-labelled ATP (Perkin Elmer, Waltham, MA), 10 μM cGMP (Calbiochem) and PKG reaction buffer as described previously [15] in a total reaction volume of 25 μL for 15 minutes at 30°C. The reactions were incubated for 30 minutes at 30°C, then terminated by adding 10 μ L Laemmli buffer and denaturing at 95°C for 3 minutes. Entire samples were separated by 10% SDS-PAGE, and the gel was stained with Coomassie blue, fixed, and dried for autoradiography with exposure on Kodak BioMax XAR film. For mass spectrometric analysis, samples were prepared as described above, except with 200 μM cold ATP in place of the radiolabeled ATP. The samples were incubated at 30°C for 60 minutes before terminating the reaction by boiling and subjecting the samples to 10% SDS-PAGE for subsequent mass spectrometry preparation and analysis.

### Establishment and analysis of stable BHK-21 expressing PKG cell line

BHK-21 cells in a 6-well plate were transfected with 1.5 μg of pCI-Neo DNA plasmid expressing human PKGIβ-Flag or empty vector pCI-Neo DNA plasmid to generate BHK + PKG and BHK + EV cells, respectively. Cells were incubated for 48 h in DMEM supplemented with 10% HI-FBS, 100 U/mL penicillin and 0.1 mg/mL streptomycin. Transfected cells were then maintained with 800 μg/μL neomycin (Invitrogen). Cells were lysed by freeze/thaw cycles for further analysis. The expression of PKG was analyzed by Western blot analysis using monoclonal mouse α-Flag (Sigma) and mouse monoclonal α-actin (Calbiochem). PKG activity was confirmed using the CycLex cyclic GMP dependent protein kinase assay kit (MBL International, Woburn, MA) according to the manufacturer’s protocol.

### Immunoprecipitation assays

HEK293T cells were transfected with DNA constructs using the TransIT-LT1 transfection system (MirusBio, Madison, WI). Cells in T-75 flasks were transfected with 15 μg of total DNA (co-transfection of NS5 and PKG, NS5 alone, PKG alone) or mock-transfected (no DNA). Cells were harvested by scraping at 48 hours post-transfection and lysed by homogenization in 1mL lysis buffer (50 mMTris-CL (pH8.0), 75 mMNaCl, 75 mMKCl, 4 mM MgCl_2_, 1% NP-40) containing 1X Halt Protease Inhibitor Cocktail (Pierce). 500 μg of total protein from the lysate was subjected to immunoprecipitation using 10 μg of α-Flag (mouse monoclonal antibody, Sigma) in the presence of Protein G agarose beads (Invitrogen). The beads were boiled in Laemmli sample buffer to elute the precipitated proteins. The precipitated protein samples were separated on 10% SDS-PAGE gels and transferred to polyvinylidenedifluoride (PVDF) membranes. Membranes were incubated with the following antibodies: custom-made rabbit polyclonal α-WNV MTase serum to detect WNV NS5 and WNV MTase (1:1000; Covance), α-Flag to detect PKG (mouse monoclonal antibody (1:500, Sigma) or rabbit polyclonal antibody (1:2000, GenScript, Piscataway, NJ)), mouse monoclonal antibody against actin (1:1000; Calbiochem), and α-HA to detect WNV RdRp and LGTV NS5 (custom monoclonal antibody from mouse serum; 1:20, from hybridoma clone 12CA5).

### *In vitro* binding assay

DNA encoding human PKGIβ in pCI-Neo (or an empty pCI-Neo plasmid for negative control) was used in a TNT reaction with ^35^S-labeled methionine (Perkin Elmer, Waltham, MA) to produce radiolabeled PKG protein. MTase proteins were expressed in *E. coli* using the pQE-30His bacterial expression system (Qiagen). The His-tag purification of MTases was done with the Ni-NTA beads as described above, but the protein was not eluted from the beads. Binding reactions consisting of 3 μL Ni-NTA beads (no protein), 10 μL Ni-NTA beads from His-purification procedure (attached MTase protein), and 10 μL TNT-produced sample were assembled and incubated overnight in co-IP lysis buffer (described above). Beads were washed, then boiled to elute bound proteins. The samples were separated by 10% SDS-PAGE. The gels were stained with Coomassie, fixed, dried, and the presence of PKG was visualized by autoradiography.

### WNV NS5 replicon mutagenesis, RNA transcription, transfection, and replication analysis

WNV replicon RNA (wild-type and αE mutant WNV replicon) was linearized with XbaI and *in vitro* transcribed with themMessagemmachine kit (Ambion, Carlsbad, CA) (manufacturer’s protocol). RNA was electroporated into 8 × 10^6^ HEK293T cells and plated in 12-well plates as previously described [34]. The medium was removed and cells were lysed at various time points post-transfection in 250 μL 1X RenillaLysis Buffer (Promega). The lysates were frozen at −80°C prior to the assay, and the total protein in the lysates was quantified with the Coomassie Plus (Bradford) Assay Kit (Pierce). To determine the luciferase activity, 10 μg of total protein from the lysate was added to 90 μL of the Renilla luciferase substrate. Luciferase activity was detected using a Glomax 20/20 Luminometer (Promega) and measured in relative light units (RLUs) per 10 μg of total protein. Triplicate wells were transfected for each sample at each time point.

### Methyltransferase reactions

Capped RNA substrates: Capped RNA substrates for methyltransferase assays were generated as described by Ray *et al*. [8]. Briefly, capped RNA substrates representing the first 190 nucleotides of the 5′-terminal WNV genome were *in vitro* transcribed from PCR products generated with primer **1** (5′-CAG *TAA TAC GAC TCA CTA TTA* GTA GTT CGC CTG TGT GAG CTG ACA AAC TT-3′ and primer **2** (5′-TCT TCA GTC CAA TCA AGG ACA ACA CGC-3′), primer **1** containing a bacteriophage T7 class II ϕ2.5 promoter (italicized) that allows ATP initiated transcript using T7 RNA polymerase [35]. *In vitro* WNV RNA transcripts were capped in the presence or absence of SAM with (α-^32^P) G*TP and Vaccinia virus capping enzyme (CellScript, Madison, WI) to generate G*pppA-RNA and m7G*pppA-RNA. The resulting capped RNA was purified through a G-50 column (GE Healthcare, Waukesha, WI) and subsequently used in methylation assays.

Cap methylation assays: The N-7 methylation reaction was performed at 30°C for 15 min in N7 buffer (50 mM MES, pH6.0, 1 mM DTT, 1 mM MgCl2, and 6 mMKCl) containing 5000 CPM G*pppA-WNV RNA, 0.5 mg purified wild-type or αE mutant methyltransferase and 100 mm SAM. The 2′-O methylation reaction was performed at 30°C for 45 minutes in 2′-O buffer (50 mm Tris, pH8, 1 mm DTT, 1 mmmgCl2, and 6mm KCl) containing 5000 CPM m7G*pppA-WNV RNA, 0.5 mg purified wild-type or αE mutant WNV methyltransferase and 100 mM SAM. Control m7GpppA-RNA and m7GpppAm-RNA was prepared using Vaccinia virus capping enzyme or Vaccinia virus Cap 1 methyltransferase (Cellscript, Madison, WI) respectively, following the manufacturers protocol. Methylation reactions were digested for one hour at 68°C with Nuclease P1 (US Biological, Swampscott, MA) and analyzed by polyethyleneimine cellulose thin layer chromatography in 0.2M ammonium sulfate.

### Statistical analysis

A two-tailed unpaired Student’s *t* test (GraphPad, San Diego, CA) was used to test the differences between two groups (e.g. comparison of the replication of individual αΕ mutant replicon to replication of the wild-type WNV replicon, comparison of PKG activity between two stable cell lines). A two-tailed Mann–Whitney U test was used to test the differences between two groups in WNV infections. A *P* value ≤ 0.05 was considered statistically significant.

## Competing interests

The authors declare that they have no competing interests.

## Authors’ contributions

JAK planned and carried out all (co-)immunoprecipitation assays, in vitro kinase assays, and replicon assays, prepared figures, and drafted the manuscript. DB performed mass spectrometry analysis. MS and RK provided WNV MTase and PYL and KB performed WNV infections and plaque assays. SF and BW performed MTase assays with MTase provided by JAK. KB, RK and RS helped with overall planning and drafting of the manuscript. All authors read, contributed feedback, and approved the manuscript.

## Supplementary Material

Additional file 1 Viral yields from infected BHK cells with and without PKG. Click here for file

## References

[B1] Nene VWJ Lawson D Haas B Kodira C Genome sequence of Aedes aegypti, a major arbovirus vector Science 2007 10 5832 1718 1723 10.1126/science.1138878 17510324 PMC2868357

[B2] Vasilakis NWS The history and evolution of human dengue emergence Adv Virus Res 2008 10 1 76 19081488 10.1016/S0065-3527(08)00401-6

[B3] Hayes EBKN Nasci RS Montgomery SP O’Leary DR Campbell GL Epidemiology and transmission dynamics of West Nile virus disease Emerg Infect Dis 2005 10 8 1167 1173 10.3201/eid1108.050289a 16102302 PMC3320478

[B4] Rosen L The natural history of Japanese encephalitis virus Annu Rev Microbiol 1986 10 395 414 10.1146/annurev.mi.40.100186.002143 2877613

[B5] Gaunt MWSA De Lamballerie X Falconar AKI Dzhivanian TI Gould EA Phylogenetic relationships of flaviviruses correlate with their epidemiology, disease association, and biogeography J Gen Virol 2001 10 8 1867 1876 11457992 10.1099/0022-1317-82-8-1867

[B6] Grard GMG Charrel RN Holmes EC Gould EA De Lamballerie X Genomics and evolution of Aedes-borne flaviviruses J Gen Virol 2010 10 1 87 94 10.1099/vir.0.014506-0 19741066

[B7] Egloff MPBD Selisko B Romette JL Canard B An RNA cap (nucleoside-2’-O-)-methyltransferase in the flavivirus RNA polymerase NS5: crystal structure and functional characterization EMBO J 2002 10 11 2757 2768 10.1093/emboj/21.11.2757 12032088 PMC125380

[B8] Ray DSA Tilgner M Guo Y Zhao Y Dong H Deas TS Zhou Y Li H Shi PY West Nile virus 5’-cap structure is formed by sequential guanine N-7 and ribose 2′-O methylations by nonstructural protein 5 J Virol 2006 10 17 8362 8370 10.1128/JVI.00814-06 16912287 PMC1563844

[B9] Ackermann MPR De novo synthesis of RNA by the dengue virus RNA-dependent RNA polymerase exhibits temperature dependence at the initiation but not elongation phase J BiolChem 2001 10 43 39926 39937 10.1074/jbc.M104248200 11546770

[B10] Leveque VJWQ RNA-dependent RNA polymerase encoded by hepatitis C virus: biomedical applications Cell Mol Life Sci 2002 10 6 909 919 10.1007/s00018-002-8478-7 12169021 PMC11337505

[B11] Keating JASR Phosphorylation events during viral infections provide potential therapeutic targets Rev Med Virol 2012 10 3 166 181 10.1002/rmv.722 22113983 PMC3334462

[B12] Reed KEGA Rice CM The NS5A/NS5 proteins of viruses from three genera of the family Flaviviridae are phosphorylated by associated serine/threonine kinases J Virol 1998 10 7 6199 6206 9621090 10.1128/jvi.72.7.6199-6206.1998 PMC110437

[B13] Kapoor MZL Ramachandra M Kusukawa J Ebner KE Association between NS3 and NS5 proteins of dengue virus type 2 in the putative RNA replicase is linked to differential phosphorylation of NS5 J BiolChem 1995 10 32 19100 19106 10.1074/jbc.270.32.19100 7642575

[B14] Morozova OVTN Maksimova TG Bachvalova VN Matveeva VA Kit YY Phosphorylation of tick-borne encephalitis virus NS5 protein Virus Res 1997 10 1 9 15 10.1016/S0168-1702(96)01433-5 9178492

[B15] Bhattacharya DM Best SM Perera R Kuhn RJ Protein kinase G phosphorylates mosquito-borne flavivirus NS5 J Virol 2009 10 18 9195 9205 10.1128/JVI.00271-09 19587048 PMC2738234

[B16] Keating JABD Rund SS Hoover S Dasgupta R Lee SJ Duffield GE Striker R Mosquito protein kinase G phosphorylates flavivirus NS5 and alters flight behavior in Aedes aegypti and Anopheles gambiae Vector Borne Zoonotic Dis 2013 in press 10.1089/vbz.2012.1110 PMC3741427 23930976

[B17] Hofmann FBD Lukowski R Weinmeister P cGMP regulated protein kinases (cGK) HandbExpPharmacol 2009 10 137 62 10.1007/978-3-540-68964-5_8 19089329

[B18] Chu DMFS Thomas JW Maksymovitch EA Fosler M Corbin JD Activation by autophosphorylation or cGMP binding produces a similar apparent conformational change in cGMP-dependent protein kinase J BiolChem 1998 10 23 14649 14656 10.1074/jbc.273.23.14649 9603983

[B19] Smith JAFS Walsh KA Kumar S Corbin JD Autophosphorylation of Type Iβ cGMP-dependent protein kinase increases basal catalytic activity and enhances allosteric activation by cGMP or cAMP J BiolChem 1996 10 34 20756 20762 10.1074/jbc.271.34.20756 8702828

[B20] Alverdi VMH Versluis C Hemrika W Esposito G Van Den Heuvel R Scholten A Heck AJR cGMP-binding prepares PKG for substrate binding by disclosing the C-terminal domain J Mol Biol 2008 10 1380 1393 10.1016/j.jmb.2007.11.053 18082764

[B21] Lee JHYV Jeang KT Activation of HIV-1 expression and replication by cGMP dependent protein kinase type 1-beta (PKG1beta) Retrovirology 2007 10 91 10.1186/1742-4690-4-91 18078512 PMC2222664

[B22] Suhasini MLH Lohmann SM Boss GR Pilz RB Cyclic-GMP-dependent protein kinase inhibits the Ras/Mitogen-activated protein kinase pathway Mol Cell Biol 1998 10 12 6983 6994 9819386 10.1128/mcb.18.12.6983 PMC109281

[B23] Kroschewski HLS Butcher RE Yap TL Lescar J Wright PJ Vasudevan SG Davidson AD Mutagenesis of the Dengue virus type 2 NS5 methyltransferase domain J Biol hem 2008 10 28 19410 19421 10.1074/jbc.M800613200 18469001

[B24] Mackenzie JMKM Westaway EG West Nile virus strain Kunjin NS5 polymerase is a phosphoprotein localized at the cytoplasmic site of viral RNA synthesis J Gen Virol 2007 10 4 1163 1168 10.1099/vir.0.82552-0 17374759

[B25] Khattar EMA Kumar V Akt augments the oncogenic potential of the HBx protein of hepatitis B virus by phosphorylation FEBS J 2012 10 7 1220 1230 10.1111/j.1742-4658.2012.08514.x 22309289

[B26] Lim YSHS Hepatitis C virus NS5A protein interacts with phosphatidylinositol 4-kinase type IIIalpha and regulates viral propagation J BiolChem 2011 10 13 11290 11298 10.1074/jbc.M110.194472 PMC3064185 21297162

[B27] Bhattacharya DHS Falk SP Weisblum B Vestling M Striker R Phosphorylation of yellow fever virus NS5 alters methyltransferase activity Virology 2008 10 2 276 284 10.1016/j.virol.2008.07.013 18757072 PMC2583469

[B28] Osborne KARA Burgess E Butland S Shaw RA Coulthard A Natural behavior polymorphism due to a cGMP-dependent protein kinase of Drosophila Science 1997 10 5327 834 836 10.1126/science.277.5327.834 9242616

[B29] Ben-Shahar YRA Sokolowski MB Robinson GE Influence of gene action across different time scales on behavior Science 2002 10 5568 741 744 10.1126/science.1069911 11976457

[B30] Lima-Camara TN BR Luz PM Castro MG Lourenco-De-Oliveira R Sorgine MH Peixoto AA Dengue infection increases the locomotor activity of Aedes aegypti females PLoS One 2011 10 3 e17690 10.1371/journal.pone.0017690 21408119 PMC3050906

[B31] Shi PYTM Lo MK Kent KA Bernard KA Infection cDNA clone of the epidemic West Nile virus from New York City J Virol 2002 10 12 5847 5856 10.1128/JVI.76.12.5847-5856.2002 12021317 PMC136194

[B32] Lim PYLK Styer LM Shi PY Bernard KA Viral pathogenesis in mice is similar for West Nile virus derived from mosquito and mammalian cells Virology 2010 10 1 93 103 10.1016/j.virol.2010.01.029 20167345 PMC2835801

[B33] Black TMCA Kilili G Ivan M Tsichlis PN Vouros P Characterization of phosphorylation sites on Tpl2 using IMAC enrichment and a linear ion trap mass spectrometer J Proteome Res 2007 10 6 2269 2276 10.1021/pr0700293 17472361

[B34] Zhang BDH Zhou Y Shi PY Genetic interactions among the West Nile virus methyltransferase, the RNA-dependent RNA polymerase, and the 5′ stem-loop genomic RNA J Virol 2008 10 14 7047 7058 10.1128/JVI.00654-08 18448528 PMC2446981

[B35] Coleman TMWG Huang F Superior 5′ homogeneity of RNA from ATP-initiated transcription under the T7 phi 2.5 promoter Nucleic Acids Res 2004 10 1 e14 10.1093/nar/gnh007 14744982 PMC373309

