# Supplementary material for: West Nile virus methyltransferase domain interacts with protein kinase G
Source: Virol J. 2013 Jul 22;10:242. doi: 10.1186/1743-422X-10-242 (PMC3725163; doi:10.1186/1743-422X-10-242)
Supplement: Additional file 1 — Viral yields from infected BHK cells with and without PKG. [file 1743-422X-10-242-S1.docx]

| WNV |  | Average Viral Replication (PFU/ml) (Standard Deviation) | |
| --- | --- | --- | --- |
| Experiment # | Hours post-infection | BHK+EV | BHK+PKG |
| 1 (shown in Fig. 3) | 24 | 2.78E5 (8.21E4) | 4.65E5 (1.39E5) |
|  | 48 | 1.70E8 (6.79E7) | 3.63E8 (6.41E7) |
|  | 72 | 5.44E5 (3.27E5) | 1.21E7 (7.50E5) |
| 2 | 24 | 1.18E5 (1.71E4) | 3.95E5 (7.14E4) |
|  | 48 | 1.14E8 (1.11E7) | 2.51E8 (2.49E7) |
|  | 72 | 4.23E7 (3.27E7) | 1.84E6 (2.47E6) |
| 3 | 24 | 3.38E5 (1.25E5) | 8.50E5 (3.11E5) |
|  | 48 | 1.93E8 (3.40E7) | 8.00E8 (1.47E8) |
|  | 72 | 5.63E3 (1.55E3) | 6.25E8 (3.43E5) |

| DENV |  | Average Viral Replication (Focus Forming Units/ml) (Standard Deviation) | |
| --- | --- | --- | --- |
| Experiment # | Hours post-infection | BHK+EV | BHK+PKG |
| 1 | 24 | 3.33 (2.89) | 41.7 (5.77) |
|  | 48 | 350 (282) | 1.59E3 (699) |
|  | 72 | 3.13E3 (3.67E3) | 1.39E3 (366) |
| 2 | 24 | 9.50E4 (2.00E3) | 4.73E5 (4.04E4) |
|  | 48 | 4.70E5 (6.08E4) | 2.97E6 (3.51E5) |
|  | 72 | 3.67E3 (208) | 6.63E5 (7.02E4) |
| 3 | 24 | 4.33E3 (5.51E3) | 2.00E5 (3.00E4) |
|  | 48 | 1.70E5 (3.00E4) | 1.80E6 (6.24E5) |
|  | 72 | 1.53E3 (305) | 3.57E5 (1.11E5) |

Caption: Viral yields from infected BHK cells with and without PKG
